# Supplementary material for: Impact of Sars-Cov-2 on access to healthcare by person with disabilities
Source: PLOS Glob Public Health. 2023 Mar 28;3(3):e0001465. doi: 10.1371/journal.pgph.0001465 (PMC10047541; doi:10.1371/journal.pgph.0001465)
Supplement: Attachment — Submitted filename: Response to Reviewers.doc [file pgph.0001465.s001.doc]

Air Force Medical Center

Air Force Base

Takoradi

Western Region

GHANA

10th February, 2023

The Academic Editor

PLOS Global Public Health

RESPONSE TO REVIEWERS

On behalf of my co-authors, I welcome the assessment report and comments of the two reviewers of the research article entitled “Impact of SARS-COV-2 on access to healthcare by persons with disabilities”. We find the comments very useful and objective as they would help shape the paper and improve its relevance to the global readership of the PLOS Global Public Health.

However, please find below our specific responses to the queries raised by the reviewers:

**Query 1.** Does this manuscript meet PLOS Global Public Health’s [publication criteria](https://journals.plos.org/globalpublichealth/s/criteria-for-publication)?

Reviewer #1: Yes

Reviewer #2: Partly

**Response to Reviewer #2**

Respectfully, this study adopted the Qualitative Design, specifically, the Phenomenological Approach (PA). Therefore, the procedures and process involved are consistent with the ethos of PA which the Institutional Review Board, University of Cape Coast, Ghana, the Social Welfare Department, Western Region, Ghana, and the Disability Association, Western Region, Ghana, approved and deemed appropriate for the study.

**Query 2.** Has the statistical analysis been performed appropriately and rigorously?

Reviewer #1: N/A

Reviewer #2: No

**Response to Reviewer #2**

As indicated above, we adopted the Qualitative Design, specifically, the Phenomenological Approach (PA), a design that does not involve the use of statistical data as is the case with Quantitative Design. Therefore, this very item is not applicable to this study design, please.

**Query 3.** Have the authors made all data underlying the findings in their manuscript fully available (please refer to the Data Availability Statement at the start of the manuscript PDF file)?
Reviewer #1: Yes

Reviewer #2: No

**Response to Reviewer #2**

Respectfully, all data underlining the study were fully disclosed and made available as attachment to the manuscript at the time of submission. Consistent with universally acceptable standards for protecting participant identities, the responses of participants were anonymised with codes and captured in the results.

**Query 4.** Is the manuscript presented in an intelligible fashion and written in standard English?
PLOS Global Public Health does not copyedit accepted manuscripts, so the language in submitted articles must be clear, correct, and unambiguous. Any typographical or grammatical errors should be corrected at revision, so please note any specific errors here.

Reviewer #1: Yes

Reviewer #2: No

**Response to Reviewer #2**

Let me emphasis that the authors were very thorough, meticulous, and detailed in editing the manuscript to ensure it complies with standard English. Moreover, issues regarding ambiguity, typographical and grammatical errors were all addressed. On the whole, the manuscript conforms to PLOS Global Public Health copy edited manuscripts and meets internationally accepted English Language standards.

**Further Comments**

**Reviewer #1:**

**Comment 1:** It is important that data collection tool was given to scholars with disability for evaluation. However, it was not clearly described why was it conducted to scholars at university level but study was conducted in participant from general population group.

**Response**

Good observation. However, the study instrument went through a wide spectrum of assessment to make it ideal and suitable for the study. I refer the reviewer to Page 7, “Instrument and Ethical Consideration”, which provided detailed information on all the concerns raised. All key actors in the setting where the study was conducted made significant inputs into the instrument used for the study. For your guidance, please find below quote from page 7 of the manuscript:

The initial prepared instrument was reviewed by the regional chairman of Ghana Federation of Disability Organization (GFDO). The instrument was then given to two physically disabled university students (in wheel chair), a Senior Lecturer in Special Education at the Department of Educational Psychology, University of Cape Coast (UCC), for further assessment.

**Reviewer #1:**

**Comment 2:** In introduction section author mentioned that “The plights of PwDs are vast and deep, for example, PwDs have often been sidelined from health information, and mainstream health provision, including access to goods and services”. It was not explicitly indicated if it is expert opinion or a finding from previous studies of which could be referenced.

**Response**

Good observation. The thoughts expressed there were extracted from reference [9] on the main reference list. This will be corrected in the edited copy of the manuscript.

**Reviewer #1:**

**Comment 3:** Nevertheless, apart from loss of income that was tremendously noted during COVID-19 pandemic, how did you able to describe difference existing/existed between pre-COVID-19 and during COVID-19 era (what are the baseline? And reference) for the following mentioned factors:-

• Stigma and discrimination.
• Cost and availability of transport
• Hospital environment and equipment
• Poor attitude of health staff
• Poor communication
• Handwashing and sanitizing facilities
• Unsuitable washroom
• Cost of health care
• Poor communication
• Registration and renewal of NHIS card

**Response**

Good observation. However, it is imperative to emphasize that the challenges faced by PwDs in accessing essential healthcare services in hospitals, before the SARS-COV-2 outbreak in Ghana, are firmly established in literature and discussed in the introduction of this manuscript. Moreover, to avoid needless repetition in the discussion and be specific to the SARS-COV-2 related findings, the authors adopted an approach that summarized the pre-SARS-COV-2 situation. Hence, the authors started the discussion of the results with a statement that explained the situation before SARS-COV-2 outbreak in Ghana. For your guidance, please find below quote from pages 18 and 19 of the manuscript:

Though PwDs are always faced with healthcare access barriers, it appears the situation is compounded in the wake of the SARS-COV-2 pandemic, especially in areas like stigmatization and discrimination, cost and availability of transport, poor communication and attitude of healthcare staff, hospital environment and equipment, unacceptable hygiene, lost of income and cost of healthcare, and registration and renewal of NHIS cards. The SARS-COV-2 pandemic is therefore disproportionately impacting PwDs than the general population.

**Reviewer #1:**

**Comment 4:** Kindly describe how did you managed to collect data from “deaf and dumb”?.

**Response:** Leadership of the disability group in the Western Region, Ghana, recommended a professional sign language interpreter who assisted during the interview with the “Deaf and Dumb”. This professional interpreter has been dealing with the “Deaf and Dumb” in the Western Region, Ghana, for years and has deep understanding of how to communicate with this group. I refer you to page 8 of the manuscript, “Instrument and Ethical Consideration”, which indicates the use of a professional sign language officer. For your guidance, please find below quote from page 8 of the manuscript:

“A professional sign language interpreter, recommended by GNAD in Takoradi, volunteered and assisted in the data collection”.

**Reviewer #1:**

**Comment 5:** As commented before, there is paucity of reference on how the condition was before the pandemic.

**Response**

Good observation. However, it is imperative to emphasize that the challenges faced by PwDs, in accessing essential healthcare services in hospitals, before the SARS-COV-2 outbreak in Ghana, is firmly established in literature and discussed in the introduction of this manuscript. Moreover, to avoid needless repetition in the discussion and be specific to the SARS-COV-2 related findings, the authors adopted an approach that would summarize the pre-SARS-COV-2 situation. Hence, the authors started the discussion of the results with a statement that explained the situation before SARS-COV-2 outbreak in Ghana. For your guidance, please find below quote from pages 18 and 19 of the manuscript:

Though PwDs are always faced with healthcare access barriers, it appears the situation is compounded in the wake of the SARS-COV-2 pandemic, especially in areas like stigmatization and discrimination, cost and availability of transport, poor communication and attitude of healthcare staff, hospital environment and equipment, unacceptable hygiene, lost of income and cost of healthcare, and registration and renewal of NHIS cards. The SARS-COV-2 pandemic is therefore disproportionately impacting PwDs than the general population.

**Reviewer #1:**

**Comment 6:** Samples size has been presented as only limitation.

How about socioeconomic status; they have nothing to do with participant responses? (Or challenges they encountered during COVID-19 pandemic?).

**Response 1:** Good comment. Generally, Qualitative Designs involve comparatively smaller sample sizes which limit their generalisability. Apart from this, it was observed during the interview that some participants expressed strong emotions, which could influence their responses and for that matter the interpretations therefrom. Please, for your guidance, find a quote from the limitations on the emotions:

“Again, some respondents were a bit emotional in their responses to some questions, especially regarding attitude of healthcare staff”.

**Response 2:** However, upon a careful read of your comments, I have the understanding that your reference to “socioeconomic status” was more about the barriers faced by PwDs in accessing healthcare during the SARS-COV-2, and NOT really the limitations of the study. On socio-economic, the current study reported “cost and availability of transport, loss of income and cost of healthcare, and registration and renewal of NHIS cards” as factors that influenced access to healthcare during the SARS-COV-2 period, and these are all socio-economic factors. Therefore, your concerns about socio-economic factors have been properly reported, though not specifically christened as socio-economic factors. Thus, this cannot be a limitation to the study.

**Reviewer #2:**

**Comment 1:** It will be difficult to popularize the results found. Why not increase the sample size before wanting to publish?

**Response:** I appreciate your comments on this. However, it is important to draw your attention to the fact that this is a Qualitative Design, and a sample size of 17 is more than adequate for a study of this nature. Moreover, 17 respondents allowed the authors to have detailed interviews with each participant which elicited the kind of information required for the study. The decision to go for a sample of 17 was consistent with literature, especially with Qualitative Design. There are many Qualitative Studies that used sample sizes ranging between 5-10 participants and published in top class journals. It would interest you to note, that there are studies that used even single cases, and deemed to be good enough for publication. I refer you to some authorities in Qualitative Studies that deal with sample sizes and how saturation is attained, Cousins and Young (2014), Hennink, Kaiser, and Marconi (2017), Malterud, Siersma, and Guassora (2016), Namey and McKenna (2017), Sim, Saunders, Waterfield, and Kingstone (2018). Every study has a focus and orientation and so does this current study. Thus consistent with literature, the authors were deliberate on the number that would help elicit the issues about this group. If our interest was in numbers, we would have adopted a Quantitative Design. Thank you.

**Reviewer #2:**

**Comment 2:** Furthermore, I would like to know the added value of the study. Everything that is put in the article, we already know. People with disabilities have always been rejected in our communities.

**Response:** Once again, we respect your comments. However, we wish to emphasise that the study was looking at how already existing barriers to access to healthcare by PwDs were influenced by the SARS-COV-2 pandemic. The study was conducted during the period of the SARS-COV-2 outbreak in Ghana and sought to establish the lived experiences of PwDs around the time. It would interest you to note that this was the first study conducted in Ghana and among the very few in Africa, if not the world, that investigated healthcare access barriers among PwDs through the lens of SAR-COV-2. The authors may also want to pose a couple questions to help drive home the purpose of this study.

Is it logical to conclude that because PwDs in other countries and continents reported difficulties in access to healthcare, no further research should be conducted in that area, even if the findings affirm existing studies? Do PwDs in Ghana also have a right to have the world know about how they navigated the novel hydra headed global pandemic? Is the contention here all about publication but also to give a voice to a group (PwDs) that had to endure a double burden of existing barriers and SARS-COV-2? Do they (PwDs in Ghana) have a right to share their experiences during this apocalyptic period which even the general population dreaded? It would interest you to note that through this study, some interventions were made to help empower PwDs in the study area better cope with the pandemic.

Meanwhile, there are two main approaches to conducting research, the Quantitative Design and Qualitative Design. The Mixed Design comes in as hybrid of the two main designs. None of the two main approaches is superior to the other and their application depends on the context. For your information, most universities in Ghana strongly encourage their PhD Candidates to adopt the Qualitative Design, given our unique socio-cultural characteristics.

Therefore, what is new, what is innovative, and the novelty of this study lie in the fact that it is surely the first study conducted in Ghana, if not Africa, that investigated how PwDs accessed essential healthcare services during the period of the SARS-COV-2.

**Reviewer #2:**

**Comment 3:** What innovative interventions would you recommend to correct this?

**Response:** The study confirmed existing barriers and recommendations, but recommended the need to recommit to accelerating efforts towards eliminating all challenges PwDs face in Ghana. Especially, minded by the universal health coverage mantra in the SDG. For your guidance, a quote from page 23 of the manuscript confirms this recommendation:

“Therefore, the need to identify and eliminate the access barriers to quality healthcare services for PwDs, especially during periods of global crisis such as the SARS-COV-2 pandemic, is obvious imperative”.

Sincerely,

… **SIGNED**.…

BOTHA, NKOSI NKOSI

(Corresponding Author)
